# Using Extracted Sugars from Spoiled Date Fruits as a Sustainable Feedstock for Ethanol Production by New Yeast Isolates

**DOI:** 10.3390/molecules29163816

**Published:** 2024-08-11

**Authors:** Georgia Antonopoulou, Maria Kamilari, Dimitra Georgopoulou, Ioanna Ntaikou

**Affiliations:** 1 Department of Sustainable Agriculture, University of Patras, 2 Georgiou Seferi St., GR-30100 Agrinio, Greece; geogant@upatras.gr; 2 Institute of Chemical Engineering Sciences (FORTH/ICE-HT), Stadiou, GR-26504 Patra, Greece; 3 Department of Plant Protection Patras, Institute of Industrial and Forage Crops, Hellenic Agricultural Organization ‘DIMITRA’, GR-26442 Patras, Greece; 4 Health Faculty, Metropolitan College, Campus of Patras, 50 Ermou St., GR-26221 Patra, Greece; mkamilari@mitropolitiko.edu.gr; 5 Department of Chemical Engineering, University of Patras, GR-26500 Patra, Greece; demie.geo1999@gmail.com; 6 Department of Civil Engineering, University of Patras, GR-26500 Patra, Greece

**Keywords:** bioethanol, spoiled date fruits, food waste valorization, response surface methodology, sugar extraction, fermentation optimization, fed batch, *Saccharomyces cerevisiae*, *Zygosaccharomyces rouxii*, *Meyerozyma guilliermondii*

## Abstract

This study focuses on investigating sugar recovery from spoiled date fruits (SDF) for sustainable ethanol production using newly isolated yeasts. Upon their isolation from different food products, yeast strains were identified through PCR amplification of the D1/D2 region and subsequent comparison with the GenBank database, confirming isolates KKU30, KKU32, and KKU33 as *Saccharomyces cerevisiae*; KKU21 as *Zygosaccharomyces rouxii*; and KKU35m as *Meyerozyma guilliermondii*. Optimization of sugar extraction from SDF pulp employed response surface methodology (RSM), varying solid loading (20–40%), temperature (20–40 °C), and extraction time (10–30 min). Linear models for sugar concentration (R1) and extraction efficiency (R2) showed relatively high R^2^ values, indicating a good model fit. Statistical analysis revealed significant effects of temperature and extraction time on extraction efficiency. The results of batch ethanol production from SDF extracts using mono-cultures indicated varying consumption rates of sugars, biomass production, and ethanol yields among strains. Notably, *S. cerevisiae* strains exhibited rapid sugar consumption and high ethanol productivity, outperforming *Z. rouxii* and *M. guilliermondii*, and they were selected for scaling up the process at fed-batch mode in a co-culture. Co-cultivation resulted in complete sugar consumption and higher ethanol yields compared to mono-cultures, whereas the ethanol titer reached 46.8 ± 0.2 g/L.

## 1. Introduction

The drive for sustainability and the need to reduce reliance on fossil fuels has spurred considerable research into developing renewable energy sources, with a particular focus on bioethanol production as a viable alternative to traditional petroleum-based transportation fuels [1]. Ensuring sustainable fermentation through the exploitation of renewable and low-cost carbon sources is imperative, such as through different agricultural residues and food wastes that are generated throughout the food production chain [2]. Among them, spoiled date fruits (SDF), a readily available and underutilized agricultural waste product, have emerged as a promising avenue for various fermentations [3,4]. SDF, often discarded, represent a rich source of fermentable sugars that can be effectively harnessed for bioethanol production, and as such, there is no need for applying the complex and, in many cases, costly chemical pretreatment that is necessary for other agricultural wastes [5,6,7]. Extracting and converting these sugars into bioethanol, however, poses several technical challenges that must be addressed through the optimization of various processing steps. 

The recovery of soluble sugars via warm water is a simple and cost-effective method that has proven to be effective for various types of biomass aiming at different end-use applications [4,8,9]. In the current study, for the optimization of the extraction process, response surface methodology (RSM) based on Box–Behnken statistical experimental design (BBD) was used. RSM is a collection of statistical and mathematical techniques useful for modeling and analyzing problems in which a response of interest is influenced by several variables. And it is extremely useful to reduce the number of experiments required for the optimization of a process, i.e., cost and time, without losing the reliability and reproducibility of the results [10]. BBD was designed for response optimization as a more efficient alternative to central composite design (CCD), the most commonly used response surface-designed experiment. BBD is based on three-level incomplete factorial designs, and it typically requires fewer experimental runs than a full factorial or CCD and has no extreme points, avoiding combinations of all factors at their high or low levels, which can be beneficial for also avoiding outlier responses or non-practical conditions [11].

Another critical component for efficient and sustainable bioethanol production is the strategic selection and deployment of novel yeast strains, both in mono-culture and co-culture configurations, which can efficiently ferment different sugar profiles even at high substrate concentrations [12,13]. Leveraging the capabilities of these microorganisms, whether through natural selection or genetic engineering, holds the key to unlocking the full potential of wastes as feedstocks for sustainable bioethanol production. The utilization of wild-type yeast strains that can naturally consume a broad range of sugars may offer a cost-effective and viable alternative that avoids the complexities and regulatory hurdles associated with genetically modified organisms [14]. The identification of new isolates for bioethanol production may offer several advantages over the use of commercial strains, whether genetically modified or not. Indeed, the genetic diversity of naturally occurring wild yeast strains is vast, and differences in fermentation performance can be observed even at the strain level, as phenotypic characteristics can quickly adapt to natural environments [15]. Furthermore, wild strains can be isolated from a variety of environments that share similarities with the intended feedstocks, allowing for easier adaptation to targeted substrates such as different types of wastes, thus resulting in higher yields and productivities as well as improved tolerance to potential inhibitors.

In this context, the present study investigated the following research questions: What are the critical parameters for the most efficient aqueous extraction of sugars, and what are the margins for optimizing the process? How can high ethanol yields and titers be achieved through the appropriate combination of wild yeast strains and fermentation methods? And finally, are SDF a suitable and promising substrate for bioethanol production after optimizing their sugar extraction? By answering the above questions, this study sheds light for the first time on the exploitation of SDF sugars for bioethanol production, which has been scarcely studied to our knowledge. 

## 2. Results

### 2.1. Identification of Yeast Strains

**Figure 1:**
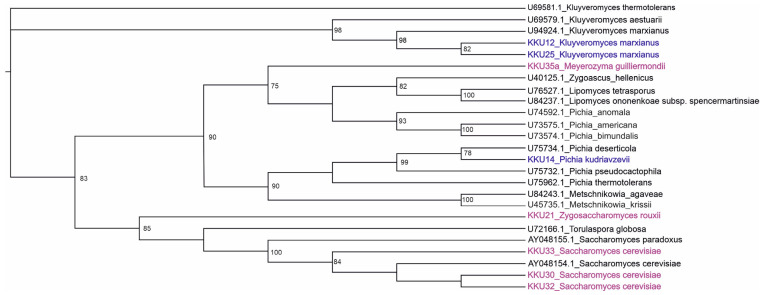
Maximum likelihood (ML) dendrogram based on the D1/D2 region of the 26S ribosomal DNA for the five samples of this study highlighted in magenta (KKU21, KKU30, KKU32, KKU33, and KKU35) and 13 yeast sequences downloaded from GenBank with their accession numbers. In blue are the samples analyzed in Ntaikou et al. [17]. Bootstrap support values over 75% are shown.

After the successful PCR amplification of the D1/D2 region and the curation of sequencing results, all sequences were compared using BLAST with the available data at GENBANK database to identify and classify the yeast isolates phylogenetically. The scores obtained were expressed as percent of the genetic identity. All obtained raw sequences were deposited in Genbank (Table 1). The isolates KKU30, KKU32, and KKU33 were identified as *Saccharomyces cerevisiae*, KKU21 as *Zygosaccharomyces rouxii*, and KKU35 as *Meyerozyma guilliermondii*. For the maximum likelihood analysis, 27 sequences were analyzed in total. The evaluation of models of nucleotide sequence evolution was performed with Moldetest at Phylemon 2.0 server [16] under the Akaike Information Criterion (AIC). The best model selected was SYM + G (−lnL = 3706.5645). The maximum likelihood phylogenetic analysis of all nucleotide sequences showed a clear grouping of the analyzed samples with their conspecific and/or closest taxonomic relatives, verifying their correct identification (Figure 1).

### 2.2. Extraction of Sugars from SDF

The recovery of sugars from the SDF pulp was studied for the loading of solids from 20 to 40% (on a wet mass basis), temperatures from 20 to 40 °C, and extraction times from 10 to 30 min, which were set as the independent variables in the BBD (A, B, and C, respectively). RSM was used to study their combinations and to determine the best conditions for optimization of water extraction in terms of sugars concentration (g/L) and efficiency (g recovered sugars/g SDF), which were the two model responses. Based on the fit of the summary analysis, linear models were suggested for both responses due to the higher R^2^ values (adjusted R^2^ for sugar concentration (R1) and for extraction efficiency (R2): 0.8619 and 0.6441, respectively) compared to the respective for quadratic (0.7957 and 0.5082) and cubic (0.6620 and 0.1506) models.

In Table 2 and Table 3, the results of ANOVA for both responses, sugars concentration, and extraction efficiency are presented. The model F-value of 7.92 implies that the linear model was significant for sugars concentration. In general, *p*-values less than 0.0500 indicate model terms are significant, while values greater than 0.1000 indicate that the model terms are not significant. In this case, A, B, and C were significant model terms, whereas the rest of the model terms were insignificant and were not counted. The lack-of-fit F-value of 0.0774 implied that the lack of fit was not significant relative to the pure error. Based on the proposed model, there was a 96.89% chance that a lack-of-fit F-value this large could occur due to noise. 

**Figure 2:**
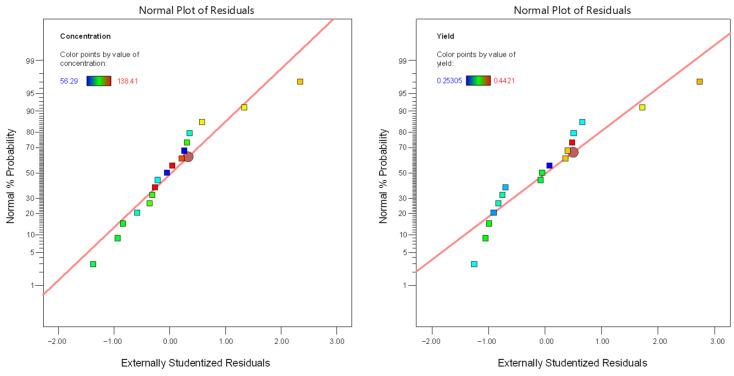
The normal plot of residuals for both responses (sugar concentration, R1; extraction efficiency, R2).

**Figure 3:**
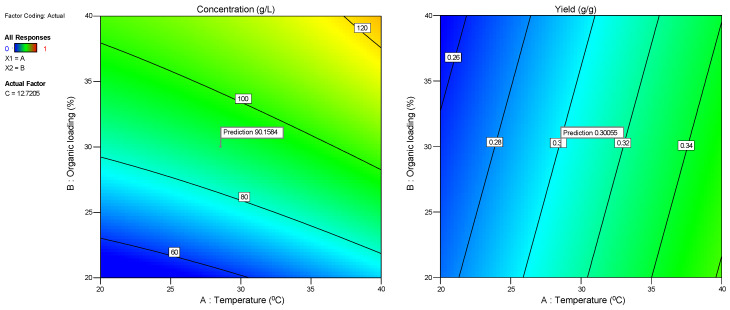
Responses R1 (sugar concentration) and R2 (extraction efficiency) for temperature and organic loading at given specific extraction time.

Regarding the linear model for extraction efficiency (R2), F-value = 10.65 (Table 3) also indicated its significance, and the relative value of *p* = 0.0062 (*p* < 0.05) indicated that the regression model was statistically significant for extraction efficiency, too. A (temperature) and C (time) were significant model terms, whereas B (organic loading) was not significant (*p* = 0.3409). The lack-of-fit F-value of 0.1608 implied the lack of fit was significant relative to the pure error. The relatively low predicted and adjusted R^2^ values (0.6038 and 0.6441, respectively) indicated that the model was not so efficient for fitting the data under specific experimental conditions, implying a moderate agreement between the predicted and experimental values for extraction efficiency. In Table 4, the linear models for sugars concentration and extraction efficiency are presented, while in Figure 2, the normal plots of residuals are depicted. The latter figure demonstrates that for both cases, the points are roughly distributed evenly above and below the regression line. In Figure 3, the relationship between responses and model prediction for the actual codes is presented. In the graphs below, the responses obtained for A and B variables are shown, while the third, C, the extraction time, was fixed at a given value of 12 min.

### 2.3. Ethanol Production from SDF Extract

#### 2.3.1. Batch Experiments with Mono-Cultures

The extraction process was optimized based on the yield of sugars from the initial biomass, indicating the relative importance of the different parameters of the experiment without taking into account the final concentration of sugars in the extracts, which was, as expected, relatively increased for the higher solids loading, ranging from 55 to 180 g/L. The effect of the substrate concentration, however, was assessed in the fermentation experiments via both mono-cultures and co-cultures. 

**Figure 4:**
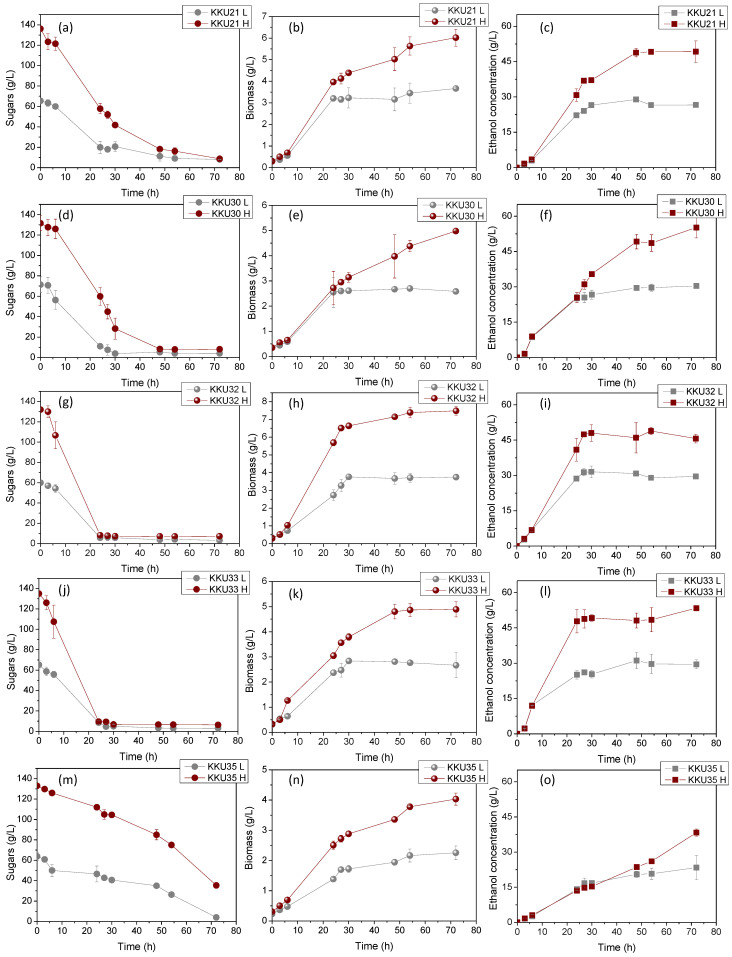
Consumption of sugars (**a**,**d**,**g**,**j**,**m**), microbial growth (**b**,**e**,**h**,**k**,**n**), and ethanol evolution (**c**,**f**,**i**,**l**,**o**) during alcoholic fermentation of SDF extracts (L, low concentration; H, high concentration) using the isolates KKU21, KKU30, KKU32, KKU33, and KKU35 in batch mode.

**Figure 5:**
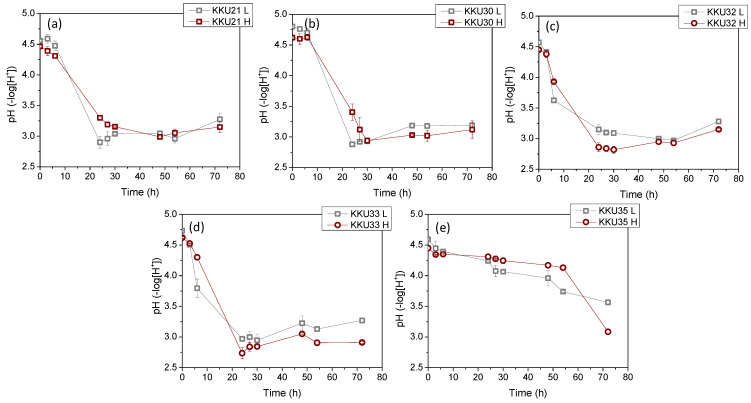
Changes of the pH values of the fermentation broth during alcoholic fermentation of SDF extracts (L, low concentration; H, high concentration) using the isolates KKU21 (**a**), KKU30 (**b**), KKU32 (**c**), KKU33 (**d**), and KKU35 (**e**), respectively, in batch mode.

Figure 4 illustrates the changes in sugar concentrations during substrate consumption (Figure 4a,d,g,j,m), microbial biomass due to substrate assimilation (Figure 4b,e,h,k,n), and ethanol during the bioconversion of sugars (Figure 4c,f,i,l,o) during the cultivation of the five isolates KKU21, KKU30, KKU32, KKU33, and KKU35 under batch conditions. Additionally, Figure 5 presents the pH variation for all cultures. As shown, the KKU21 strain almost completely consumed the sugars at both substrate concentrations by 72 h, with a rapid rate up to 30 h of incubation, decreasing thereafter. The maximum biomass concentration for the low substrate concentration experiment was 3.7 ± 0.0 g/L, achieved at 72 h of cultivation, although this value does not show a statistically significant difference from the biomass values recorded during microbial growth after 30 h of cultivation. The maximum ethanol concentration for the low substrate concentration experiment was recorded at 30 h of cultivation, reaching 28.9 ± 1.1 g/L. The maximum biomass concentration for the high substrate concentration experiment was 6.0 ± 0.1 g/L, also achieved at 72 h, where the maximum ethanol concentration of 49.2 ± 4.5 g/L was noted, although this value is not statistically greater than the values recorded after 48 h of cultivation. The pH of the cultures reached a minimum value of 2.9 ± 0.1 and 3.0 ± 0.0 for the low and high substrate concentration experiments at 24 and 48 h of incubation, respectively.

Regarding the KKU30 strain, complete sugar consumption was observed for the low substrate concentration experiment at 30 h, while 54 h of incubation were required for sugar consumption in the high substrate concentration experiment. The maximum biomass concentration for the low substrate concentration experiment was 2.6 ± 0.1 g/L, achieved at 30 h of cultivation, at which point ethanol production was also observed to reach almost its maximum (production appears to plateau), with a concentration of 26.6 ± 1.9 g/L. The maximum biomass concentration for the high substrate concentration experiment was 5.0 ± 0.0 g/L, achieved at 72 h, where the maximum ethanol concentration of 55.2 ± 4.4 g/L was noted. The pH of the cultures reached a minimum value of 2.9 ± 0.0 and 2.9 ± 0.1 for the low and high substrate concentration experiments, respectively. The KKU32 strain achieved complete substrate consumption for both concentrations studied at 24 h of cultivation, at which point the highest microbial biomass and ethanol concentrations were recorded for the low substrate concentration experiment, with values of 5.4 ± 0.0 g/L and 31.6 ± 1.5 g/L, respectively. The maximum biomass and ethanol values for the high substrate concentration experiment were 10.7 ± 0.2 g/L and 48.9 ± 1.6 g/L, respectively. The pH of the cultures reached a minimum value of 3.0 ± 0.0 and 2.8 ± 0.0 for the low and high substrate concentration experiments at 54 and 30 h of cultivation, respectively. For the KKU33 strain, complete sugar consumption was observed at 30 for both the low and high substrate concentration experiments. The maximum biomass concentration for the low substrate concentration experiment was 2.8 ± 0.1 g/L, while for the high substrate concentration experiment, it was 4.9 ± 0.3 g/L, with maximum ethanol concentrations of 31.1 ± 3.0 g/L and 53.4 ± 0.5 g/L, respectively. The pH of the cultures reached a minimum value of 2.9 ± 0.0 and 2.8 ± 0.0 for the low and high substrate concentration experiments, respectively. Finally, for the KKU35 strain, complete sugar consumption was observed for the low substrate concentration experiment and partial sugar consumption for the high substrate concentration experiment at 72 h of cultivation, at which point the maximum microbial biomass and ethanol values were also observed for both substrate concentrations studied. Specifically, the maximum biomass values were 2.3 ± 0.2 g/L and only 4.0 ± 0.2 g/L for the low substrate concentration experiment, and the maximum ethanol values were 23.4 ± 5.2 g/L and 38.3 ± 1.5 g/L for the high substrate concentration experiment.

On a comparative basis, it can be stated that in all cases, the increase in biomass and ethanol appears to be satisfactorily related to the substrate consumption pattern. That is, both microbial growth and ethanol production aligned with glycolysis, as expected based on the primary metabolism of yeasts. The pH variation exhibited a similar pattern for four out of the five strains studied, with an initially sharp decreasing trend up to 24 h of cultivation as ethanol accumulated, followed by a slightly increasing trend. Notably, differences were also observed in some cases concerning the pH variation pattern, which depends on the concentrations of metabolic products as well as nitrogen assimilation during microbial biomass production [12].

**Figure 6:**
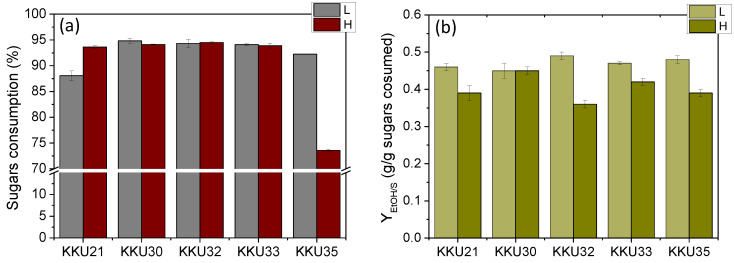
Comparative overall sugar consumption (**a**) and maximum ethanol yields (*Y_EtOH_*) (**b**) estimated during alcoholic fermentation of SDF extracts (L, low concentration; H, high concentration) using the isolates KKU21, KKU30, KKU32, KKU33, and KKU35, respectively, in batch mode.

To better compare the five strains, substrate consumption rate (*r_S_*) and ethanol production rate (*r_EtOH_*) were calculated as well as the microbial biomass yields (*Y_X_*_/*S*_), ethanol yields (*Y_EtOH_*), and the percentage of final sugar consumption. This was carried out by considering the time point at which consumption and production appeared to plateau in each case. The results are summarized in Table 5, which shows significant differences in ethanol (*Y_EtOH_*) and biomass production yields (*Y_X_*_/*S*_) as well as in the rates of ethanol production (*r_EtOH_*) and sugar consumption. For direct comparison, the yields are also presented graphically in Figure 6.

Comparing all five yeast strains studied, it was observed that the strains KKU32 and KKU33 exhibited the highest growth and ethanol production rates, indicating they are faster compared to the other strains, followed by strain KKU30. Notably, based on molecular analysis, these three strains were identified as belonging to the genus *Saccharomyces cerevisiae*, a fermentative organism known for generally high biomass yields under both aerobic and anaerobic conditions [18]. Strains KKU21 and KKU35 showed significantly lower growth and ethanol production rates, with strain KKU35 being the slowest of all. According to molecular analysis, KKU21 was identified as *Zygosaccharomyces rouxii* and KKU35 as *Meyerozyma guilliermondii*.

The overall sugar consumption was the same for all strains, both at low and high substrate concentrations, reaching 95% except for strain S35, which did not manage to consume the provided sugars in the high concentration experiment within 72 h of incubation. Biomass yields ranged between 0.34 and 0.56 g biomass/g sugars, except for strain KKU32, which achieved a yield of 0.62 g biomass/g sugars, corresponding to a final concentration of over 7 g/L.

Regarding the effect of substrate concentration on ethanol yield, it appeared extremely high for all strains in low substrate concentration experiments, ranging from 0.45 g ethanol/g sugars to 0.48 g ethanol/g sugars, corresponding to a fermentation efficiency (*FE*) of 88–94% of the maximum theoretical yield. However, ethanol yields in the high substrate concentration experiments were significantly reduced to 0.35–0.43 g ethanol/g sugars, corresponding to a fermentation efficiency of 71–74% of the maximum theoretical yield, except for strain KKU30, for which the yield remained the same at 0.45 g ethanol/g sugars. Thus, it appears that high substrate concentration negatively affects ethanol yield, with substrate inhibition occurring for all strains except KKU30 at an initial sugar concentration of 140 g/L.

#### 2.3.2. Scaling Up with Co-Culture

Based on the above, for the scaling up of the fermentation process in a bioreactor, the cultivation of strains KKU30 and KKU33 was chosen. The first strain was shown not to be inhibited by the high substrate concentration, while the second exhibited faster metabolism during fermentation. Figure 6 schematically illustrates the experimental results in terms of sugars consumption and *Y_EtOH_* for all strains tested. 

**Figure 7:**
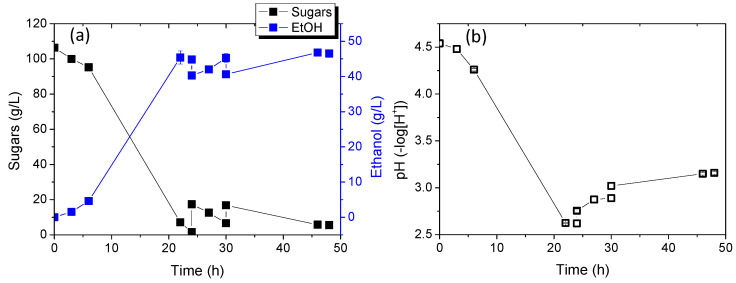
Sugar consumption and ethanol production (**a**) as well as pH change (**b**) during alcoholic fermentation of SDF extract using the co-culture of strains KKU30 and KKU33 in fed-batch mode.

Figure 7 shows the results of sugar consumption relative to ethanol production and pH changes over time. Biomass production was not continuously monitored due to challenges converting optical absorption changes to concentration, given the significant differences in calibration curves between the two yeast strains. However, biomass quantification was conducted at the end of the experiment to estimate biomass yield. As shown at 24 h of cultivation, sugar consumption was complete, with approximately 110 g/L sugars consumed. Notably, in mono-cultures, strains KKU30 and KKU33 consumed 72 g/L and 125 g/L of sugars, respectively, after 24 h. Ethanol concentration reached 45.4 ± 1.8 g/L, resulting in a yield of 0.46 ± 0.01 g ethanol/g sugars. pH significantly decreased to 2.6 ± 0.0, consistent with results from mono-cultures, where pH typically rises after 24 h.

The subsequent addition of a new substrate in two doses again led to complete sugar consumption and a slight increase in ethanol concentration, peaking at 46.8 ± 0.2 g/L. Ethanol yield from the overall process reached 0.48 ± 0.01 g ethanol/g sugars. 

Furthermore, additional substrate addition in semi-continuous operation positively impacted the process, increasing overall ethanol yield and causing a minor ethanol concentration boost. However, further experiments with larger quantities or substrate concentrations are necessary to draw clear conclusions regarding the potential for greater ethanol concentration increases. Finally, to estimate process yield from exploiting initial waste, the ethanol quantity produced from waste was calculated as Y_EtOH_/SDF, at 351.0 ± 0.01 g ethanol/kg SDF.

In conclusion, co-cultivation appears to favor the process, achieving rapid and complete sugar consumption and higher ethanol yields compared to mono-cultures. No substrate inhibition was observed, with concentrations exceeding 40 g/L, which is known in the literature as the minimum for satisfactory ethanol distillation from a fermentation mix [19].

## 3. Discussion

In general, the RSM technique has been utilized in many areas and has demonstrated its efficacy as a numerical method through its comparison to experimental studies and other numeric research [20]. There are three steps in RSM implementation; the first one involves the design of the experiment using BB or central composite, the second involves statistical and regression analysis to develop equations of the model that represent the response surface modeling; and finally, the third is the optimization of the parameters/variables carried out through model equations [21]. Based on the above, it can be concluded that the RSM model, although it resulted in a satisfactory simulation of the experimental data, was not quite efficient in optimizing the process since it resulted in a linear model in contrast to previous studies such as that of Messadi et al. [22], in which a quadratic polynomial regression model was established. In that study, the aim was to maximize the sugar yield of Tunisian date powder, also employing RSM and BBD to characterize the optimized extract from Tunisian date powder and to assess its antioxidant capacity, and the impacts of extraction time and temperature as well as the ratio between solvent and date powder on sugar extract from date powder were assessed. It should be noted though that extractions times selected were much higher (0–8 h) compared to the levels selected in the current study. 

With respect to the efficiency of the aqueous extraction of sugars from SDF slurry, it can be noted that the methodology exhibited a high recovery ratio potential. In particular, the highest yield of sugars that was achieved experimentally and was in good agreement with the prediction of the model was 0.43 g sugars/g SDF, which corresponds to approximately 84% recovery of the contained sugars of the waste (51.32 ± 0.84% sugar content). Taking also into account that the correlation of the performance with the selected factors was linear, it can be assumed that a further increase in the upper levels of factors would also lead to greater performances. At this point, though, it must be pointed out that such an attempt may hinder the practical limitation and may be applied for certain factors only, i.e., time and temperature. Indeed, the 40% solids loading that was used as the higher study point was selected because, for higher loadings, the stirring of the mixture was insufficient, whereas the subsequent separation step of the extract from the solid fraction was also quite challenging. This is in agreement with previous studies with starchy substrates, in which the solid loading could not exceed the limit of 40% due to operational challenges [12,13,23].

Besides the optimization of sugar recovery from SDF, sustainable ethanol production can be significantly enhanced by leveraging the unique benefits and characteristics of the yeast stains of *Z. rouxii*, *S. cerevisiae*, and *M. guilliermondii* that were selected for the bioconversion of SDF sugars. *Z. rouxii*, with exceptional tolerance to high sugar and salt concentrations, thrives in the osmotic-stress environment typical of spoiled dates, ensuring robust fermentation even under challenging conditions [24]. *S. cerevisiae*, well known for its high ethanol yield and efficiency in glucose fermentation, provides rapid and efficient conversion of sugars to ethanol, making it a cornerstone in bioethanol production [25]. *M. guilliermondii* brings versatility in utilizing diverse sugar substrates and can produce valuable enzymes, enhancing the breakdown of complex sugars in date fruits [23,26]. By employing these yeasts in mono-cultures or co-cultures, their complementary metabolic pathways can be harnessed to optimize fermentation efficiency, increase ethanol yields, and improve the overall sustainability of the process [27]. The specific advantages of *Z. rouxii*, *S. cerevisiae*, and *M. guilliermondii*, including their stress tolerance, metabolic capabilities, and complementary interactions, make them valuable in this biotechnological application. This approach not only maximizes the utilization of spoiled date fruits, reducing agricultural waste, but also supports renewable energy production, contributing to a more sustainable bioeconomy.

Accurate genetic identification via techniques like PCR, sequencing, and DNA barcoding are essential to accurately characterize and differentiate yeast strains, ensuring that only the most suitable ones are used. Furthermore, it allows to track and select the most robust strains that have undergone adaptive evolution to thrive in the high-sugar and stressful environments typical of spoiled dates. To this end, advanced genomic analyses can reveal synergistic interactions and pin-point genes for improved ethanol production [28]; the patterns of different yeast strains’ interaction at the molecular level can enable the design of co-cultures where each strain complements the other, leading to more efficient sugar utilization and higher ethanol yields. Understanding the genetic basis of growth and metabolism can help to adjust fermentation conditions to maintain a balanced co-culture, preventing dominance by one strain and ensuring optimal performance [29].

Based on the above, the overall conclusion drawn from the outcomes of the current study may be that by optimizing recovery methodology and deploying innovative yeast mono-cultures and co-cultures, bioethanol production can be made more sustainable, economically viable, and environmentally friendly, contributing to the overall shift towards a greener energy landscape.

## 4. Materials and Methods

### 4.1. Feedstock 

SD was obtained from the local street market of Abha city, Kingdom of Saudi Arabia. The discarded whole fruits containing seeds and skins were collected, air-dried, packed in PPT bags in batches of 2 kg, and were transported, sealed, to Greece. Contamination of the SD with insect larvae (4–6 mm, whitish) was observed in some cases, but no evident other fungal or bacterial degradation was noticed by the bare eye. Upon arrival at the laboratory, larvae and fruit seeds were manually removed, and the remaining fruits were washed with warm water and air-dried for 24 h. The air-dried fruits, containing solely the sugary mesocarp and the exocarp, were then milled in batches with a conventional grinder until the formation of a homogeneous pulp. Batches were mixed and divided into 250 mL sealed plastic cups, which were stored at −21 °C.

For the characterization of dates, one batch of homogenized pulp was used prior to freezing. The chemical characteristics of the SD pulp were as follows: total solids (TS), 68.37 ± 1.28%; humidity, 31.63 ± 1.81%; volatile solids, (VS), 96.71 ± 0.08%; ash (% TS), 2.25 ± 0.11%; pH (10% aqueous solution), 6.12 ± 0.02; sugars, 51.32 ± 0.84%; total carbohydrates, 66.33 ± 3.67%; TKN, 0.61 ± 0.04%; proteins, 3.75 ± 0.25%.

### 4.2. Extraction of Sugars from SDF

In all cases, the water extraction of sugars from SDF was performed using tap water and under constant mechanical agitation at 150 rpm. The liquid (extract) was then separated from the solids via centrifugation for 15 min at 4000 rpm. The concentration of soluble sugars was quantified in the extracts, and the recovery yield (extraction efficiency) was estimated in each case as g recovered sugars/g SDF. 

### 4.3. Experimental Design and Statistical Analysis

In this study, the experimental design of the recovery process of soluble sugars from the SDF was based on three-level three-factor BBD aiming to determine the best combination of extraction variables (factors), i.e., extraction temperature (A), organic loading (B), and extraction time (C). These were used as independent variables, while sugars concentration (g/L) and extraction efficiency (g sugars/g SDF) were selected as the responses factors (R1 and R2). Independent variables for extraction were placed at three equally spaced values (levels) and coded as −1, 0, and +1, with five replicates at the center point, giving a total of 17 experimental runs. Table 6 presents the BBD matrix used in the present study, including the three real and coded variables as well as the responses factors. 

Experimental design was performed using Design-Expert^®^ (DX, Version 12) or Stat-Ease 360^®^ software (Trial Version, 2022), and the analysis and optimization used RSM. Assessment of the variables effects, interactions, and fitness of the polynomial model equations was performed using the coefficient of determination R^2^, while the statistical significance and significance of the regression coefficients were expressed by F-test using ANOVA. 

### 4.4. Identification of Microorganisms

For the strains analyzed, total genomic DNA was extracted from ca. 10^6^ cells, using a DNeasy PowerSoil Kit (QIAGEN, Hilden, Germany) following the manufacturer’s protocol. Following DNA extraction, PCR amplification was performed targeting D1/D2 domain of 26S rDNA regions, with primers NL1 (5′-GCATATCAATAAGCGGAGGAAAAG-3′), and NL4 (5′-GGTCCGTGTTT CAAGACGG-3′) from Hashem et al., yielding a PCR product of approximately 600 bps. The D1/D2 region is a commonly used region for yeast identification: It contains variable regions interspersed with conserved regions and has proven to be informative for distinguishing between different yeast species and identifying unknown isolates [4,30]. 

PCRs were carried out in 25 μL volumes (1X PCR Master Mix: KAPA2G FAST Multiplex PCR Kit and 1 U of Hot start Taq DNA Polymerase) with 1 μL of each primer (10 μM) and 1 μL DNA template, filled to 25 μL with double-distilled water. Thermocycling included an initial denaturation step at 95 °C for 5 min, followed by 35 cycles of denaturation at 95 °C for 30 s, annealing at 52 °C for 1 min, and extension at 72 °C for 90 s, with a final extension step at 72 °C for 10 min. The PCR results were analyzed by horizontal electrophoresis in 1% agarose gel stained with GelRed^®^ (Biotium, Fremont, CA, USA) and inspected under UV light. PCR products were purified using commercially available spin columns (Macherey-Nagel). Sequencing was conducted on an AB3700 capillary sequencer (Macrogen Europe, Amsterdam, The Netherlands) using the primers of the amplification procedure. 

The resulting chromatograms were manually inspected, and the corrected sequences were subjected to multiple alignments with CLUSTAL-W v1.4 [31]. Sequences were analyzed with Gblocks’ 0.91b using the default parameters for the removal of ambiguous regions [32]. Maximum likelihood (ML) analysis was conducted with PhyML 3.0 (default parameters), and robustness was tested by bootstrap analysis with 1000 replicates [33]. 

### 4.5. Bioethanol Production

#### 4.5.1. Isolates and Pre-Cultures

The yeast strains were isolated by the dilution plate method from various food wastes and residues collected from restaurants in the Asir Region, Saudi Arabia, and were purified as described by Hashem et al. [12]. Specifically, KKU21 was isolated from spoiled cooked rice, KKU30 and KKU32 from bread, KKU33 from guava fruit, and KKU35 from mango fruit.

Pre-cultures were prepared by transferring a full bacteriological loop of yeast colonies from slant cultures (YMA medium) that were stored at 4 °C to 100 mL YMB medium in an Erlenmeyer flask (250 mL) under aseptic conditions. The flasks were capped with hydrophobic cotton and incubated at 30 °C and 150 rpm overnight until reaching OD600 nm ~1.6.

#### 4.5.2. Fermentation Experiments

Mono-cultures of the strains KKU21, KKU30, KKU32, KKU33, and KKU35 were performed in duplicate in batch mode in serum vials with a working volume of 60 mL. The vials were sealed with rubber stoppers equipped with 0.22 µm sterilized filters for CO_2_ venting and incubated at 30 °C with constant agitation at 150 rpm. For the inoculation, in all experiments, cells were harvested from each pre-culture of the isolates at 10% *v*/*v*.

Co-cultures of the strains KKU30 and KKU33 were conducted in duplicate using two identical quartz bioreactors of 1.5 total volume and 0.5 L working volume. Similarly to the mono-culture experiments, 0.22 µm sterilized filters were placed via rubber septa at the top of each for CO_2_ venting and incubation, followed at 30 °C with constant agitation at 150 rpm. For the inoculation, cells were harvested from each pre-culture of the KKU30 and KKU33 isolates at 5% *v*/*v*. As the sole carbon source for the fermentations, SDF extract of 170 g/L concentration of sugars was further diluted to ~120 g/L using distilled water to mitigate potential substrate inhibition from high sugar concentration. Moreover, substrate feeding occurred over three cycles by supplying fresh, undiluted SDF extract after 24 h and 30 h of cultivation. Specifically, after 24 h, sugar concentration was checked and found to be fully consumed, prompting the addition of 25 mL of fresh extract, followed by a 6 h incubation. At 30 h, another 25 mL of fresh extract was added, incubating until 48 h. 

For the preparation of the inocula, the estimated volume of pre-cultures was centrifuged at 4500× *g* for 15 min, and the yeast pellet was re-suspended in a solution containing KH_2_PO_4_, MgCl_2_. 6 H_2_O, and (NH_4_)_2_SO_4_ each at final concentrations of 1 g/L culture. The efficiency of alcoholic fermentation was assessed by estimating bioethanol yields in terms of carbohydrate uptake and initial feedstock bioconversion. The concentration of microbial biomass (g/L) was also quantified using the following calibration curves. KKU21, 0.7259 × OD600 nm—0.0586, R^2^ = 0.993; KKU30, 0.5953 × OD600 nm—0.0586, R^2^ = 0.990; KKU32, 0.6487 × OD600 nm—0.0412, R^2^ = 0.993; KKU33, 0.6157 × OD600 nm—0.0167, R^2^ = 0.990; KKU35, 0.4486 × OD600 nm—0.0381, R^2^ = 0.996.

To assess the efficiency of alcoholic fermentation, bioethanol yield in terms of sugars uptake (*Y_EtOH_*_/*S*_), fermentation efficiency (*FE*), and feedstock bioconversion (Y_EtOH/SDF_) were calculated according to the following equations:

(1) $$Y_{EtOH/S}\;\left(\frac{g}{g}\right)=\frac{Bioethanol\;concentration\;\left(\frac{g}{L}\right)}{Consumed\;sugars\;\left(\frac{g}{L}\right)}\;\;\;$$

(2) $$FE\;\left(\%\right)=\frac{Y_{EtOH/S}\;}{Y_{EtOH/S}\;max}\;\;\;\;$$

where *Y_EtOH_*_/*S*_ is the estimated ethanol yield from the consumed sugars, and *Y_EtOH_*_/*S*_*^max^* is the maximum theoretical ethanol yield, i.e., 0.51 g ethanol/consumed hexoses, according to the Guy–Loussac equation.

(3) $$Y_{EtOH/SDF}\;\left(\frac{g}{\mathrm{kg}}\right)=\frac{Bioethanol\;concentration\;\left(g\right)}{SDF\;\;\left(\mathrm{kg}\right)}\;\;\;$$

where *SDF* is the mass of SDF pulp that was used for the production of extract used in the fermentation.

### 4.6. Analytical Methods

TS, VS, and TKN were quantified according to standard methods [34]. Crude protein content was determined by multiplying TKN by a factor of 6.25 [35]. Total carbohydrates and soluble sugars were quantified according to DuBois et al. [36]. Ethanol was quantified via HPLC (DIONEX) using an RI detector (Shodex) and an Aminex HPΧ–87H column (Biorad) as previously described by Ntaikou et al. [23]. In brief, an isocratic method was used, with H_2_SO_4_ 0.006Ν as eluent at a flow rate of 0.6 mL.min^−1^ and at 60 °C.

## Figures and Tables

**Table 1:** Molecular identification of yeast samples using Sanger sequencing and comparing to NCBI BLAST tool at GenBank. The percentage of similarity and best match are provided.

Sample ID	Best Hit on GENBANK (Accession Number)	Similarity (%)	Genbank Accession Number
KKU21	*Zygosaccharomyces rouxii isolate* D13 (MK341573.1)	99.08	OR245523
KKU30	*Saccharomyces cerevisiae isolate* WHY-5 (MG641150.1)	99.3	OR245524
KKU32	*Saccharomyces cerevisiae strain* GV5 03 (KP250857.1)	99.17	OR245525
KKU33	*Saccharomyces cerevisiae strain* GV5 03 (KP250857.1)	99.65	OR245526
KKU35	*Meyerozyma guilliermondii strain* ML4 (MK907983.1)	98.62	OR245527

**Table 2:** Results of ANOVA for sugars concentration (R1).

Sources	Sum of Squares	DF	Mean Square	F-Value	*p*-Value *
Linear Model	9394.68	9	1043.85	7.92	0.0062 (s)
A—Temperature	1192.31	1	1192.31	9.05	0.0197
B—Organic loading	6224.49	1	6224.49	47.24	0.0002
C—Time	1742.42	1	1742.42	13.22	00083
AB	5.38	1	5.38	0.0409	0.8456
AC	7.49	1	7.49	0.0569	0.8183
BC	20.25	1	20.25	0.1597	0.7067
A^2^	11.09	1	11.09	0.0842	0.7801
B^2^	163.46	1	163.46	1.24	0.3021
C^2^	25.99	1	25.99	0.1972	0.6704
Residual	922.27	7	131.75		
Lack of Fit	50.60	3	16.87	0.0774	0.9689 (ns)

* s: significant; ns: not significant.

**Table 3:** Results of ANOVA for extraction efficiency (R2).

Source	Sum of Squares	DF	Mean Square	F-Value	*p*-Value *
Linear Model	0.0327	3	0.0109	10.65	0.0008 (s)
A—Temperature	0.0153	1	0.0153	14.97	0.0019
B—Organic loading	0.0010	1	0.0010	0.9772	0.3409
C—Time	0.0164	1	0.0164	16.01	0.0015
Residual	0.0133	13	0.0010		
Lack of Fit	0.0035	9	0.0004	0.1608	0.9890 (ns)

* s: significant; ns: not significant.

**Table 4:** Linear models for sugar concentration (R1) and extraction efficiency (R2).

Response	Final Equations in Terms of Actual and Coded Factors	
Concentration (R1)	Coded	R1 = 100.453 + 12.2081 × A + 27.8938 × B + 14.7581 × C
	Actual =	49.3692 + 1.22081 × A + 2.78938 × B + 1.47581 × C
Extraction Efficiency (R2)	Coded	R2 = 0.3398 + 0.0438 × A − 0.0112 × B + 0.0453 × C
	Actual =	0.151401 + 0.004379 × A − 0.00119 × B + 0.004528 × C

**Table 5:** Aggregate results of yields and rates of the fermentation process during alcoholic fermentation of SDF extracts (L, low concentration; H, high concentration) using the isolates KKU21, KKU30, KKU32, KKU33, and KKU35 in batch mode. *r_S_*, substrate consumption rate; *r_EtOH_*, ethanol production rate; *Y_X_*_/*S*_, microbial biomass yield, *Y_EtOH_*, ethanol yield.

Isolate	Extract Concentration	*r_S_*, g/L·h	*r_EtOH_*, g/L·h	*Y_X_*_/*S*_, g Biomass/g Sugars Cons.	*Y_EtOH_*_/*S*_, g Eth/g Sugars Cons.	Sugar Consumption, %
KKU21	L	1.05 ± 0.02	0.60 ± 0.08	0.056 ± 0.001	0.46 ± 0.01	89.1 ± 0.9
	H	1.77 ± 0.11	0.91 ± 0.02	0.045 ± 0.001	0.39 ± 0.01	93.6 ± 0.3
KKU30	L	2.25 ± 0.04	0.89 ± 0.01	0.035 ± 0.001	0.45 ± 0.02	94.8 ± 0.5
	H	2.29 ± 0.09	0.90 ± 0.14	0.038 ± 0.000	0.45 ± 0.02	94.1 ± 0.1
KKU32	L	2.87 ± 0.08	1.05 ± 0.01	0.062 ± 0.001	0.48 ± 0.01	94.3 ± 0.8
	H	4.16 ± 0.25	1.34 ± 0.06	0.058 ± 0.002	0.36 ± 0.01	94.5 ± 0.1
KKU33	L	5.86 ± 0.05	1.30 ± 0.11	0.039 ± 0.007	0.47 ± 0.00	94.1 ± 0.2
	H	4.27 ± 0.25	1.64 ± 0.29	0.035 ± 0.002	0.43 ± 0.01	93.9 ± 0.5
KKU35	L	0.83 ± 0.02	0.32 ± 0.01	0.034 ± 0.003	0.48 ± 0.01	92.2 ± 0.1
	H	1.36 ± 0.05	0.53 ± 0.05	0.038 ± 0.013	0.39 ± 0.02	73.6 ± 0.2

**Table 6:** BB experimental design and response factors for sugars concentration and yields. The three levels (−1, 0, and 1) of factors: (a) A (extraction temperature) represented 20, 30, and 40 °C; (b) B (solids loading) represented 20, 30, and 40%; and (c) C (extraction time) represented 10, 20, and 30 min, respectively.

Run	A	B	C	Concentration, g/L	Yield, g Sugars/g SDF
1	0	1	1	143.10	0.373
2	0	1	−1	113.58	0.282
3	1	−1	0	84.66	0.395
4	−1	1	0	116.23	0.284
5	−1	0	1	103.10	0.341
6	0	0	0	100.45	0.339
7	0	0	0	100.45	0.339
8	0	0	0	100.45	0.339
9	−1	−1	0	60.45	0.308
10	1	0	−1	97.80	0.338
11	0	0	1	115.21	0.385
12	0	0	0	100.45	0.339
13	1	0	1	127.32	0.428
14	1	1	0	140.45	0.371
15	0	−1	1	87.31	0.397
16	0	−1	−1	57.8	0.306
17	−1	0	−1	73.58	0.250

## Data Availability

The data are available upon request.
